# Sustainable Energy Storage Systems: The Promise of Biomass-Derived Carbon Materials for High-Performance Supercapacitors

**DOI:** 10.3390/nano16090524

**Published:** 2026-04-26

**Authors:** Aigerim R. Seitkazinova, Muhammad Hashami, Meruyert Nazhipkyzy, Roza G. Abdulkarimova, Zhanar B. Kudyarova, Aigerim G. Zhaxybayeva, Saltanat S. Kaliyeva, Balken T. Kuderina, Bakhytzhan T. Lesbayev

**Affiliations:** 1 Department of Chemical Physics and Material Science, Al-Farabi Kazakh National University, 71 Al-Farabi Ave., Almaty 050038, Kazakhstan; aiko3126@mail.ru (A.R.S.); roza.abdulkarimova@kaznu.edu.kz (R.G.A.); zhanar.kudyarova@kaznu.edu.kz (Z.B.K.); bakytzhan.lesbayev@kaznu.edu.kz (B.T.L.); 2 Institute of Combustion Problems, Bogenbai Batyr Street 172, Almaty 050012, Kazakhstan; 3 Department of Chemistry, Education Faculty, Mirwais Khan Nika University Zabul, Qalat 4001, Afghanistan; mg.hashami2010@gmail.com; 4 Department of Chemistry, Kazakh National Women’s Teacher Training University, Gogol Str., 114 k1, Almaty 090000, Kazakhstan; 5 Department of Chemistry, Faculty of Natural Sciences, L.N. Gumilyov Eurasian National University, Kazhymukan Str. 11, Astana 010008, Kazakhstan; zhaxybayeva_ag_1@enu.kz; 6 Higher School of Natural Sciences, Astana International University, Astana 010008, Kazakhstan; saltanat_kalieva@aiu.edu.kz; 7 “Water Quality” Scientific Research Laboratory, NJSC “Kokshetau University Named After Sh. Ualikhanov”, Kokshetau 020000, Kazakhstan; b.kuderina@shokan.edu.kz

**Keywords:** biomass-derived carbon, food waste, energy storage, pseudocapacitors, EDLC, hybrid capacitors

## Abstract

The rapid demand for sustainable and efficient energy storage solutions has prompted the pursuit of eco-friendly electrode materials. Biomass-derived carbons from food waste offer a promising pathway to meet this need by combining waste valorization, environmental benefits, and high electrochemical performance. This review highlights that food waste biomass is an effective and inexpensive source of precursors for producing high-performance carbon materials for supercapacitors. Food waste, which includes fruit peels and vegetable residues, cereal husks, and oilseed residues, is a good source of lignocellulosic components, heteroatoms, and structural features that determine the electrochemical characteristics of the derived carbons. These wastes produce hierarchically porous carbons with high surface areas (>1500 m^2^ g^−1^) on pyrolysis and activation that provide superior ion transport, wettability and pseudocapacitive behaviour. Their electrochemical performance includes capacitances up to 520 F g^−1^ and energy densities of 35–70 Wh kg^−1^ in optimized systems, particularly under extended voltage windows or in hybrid supercapacitor configurations, and high cycling stability is equal to or even better than traditional carbons such as activated carbon and graphene. Additionally, biomass valorization contributes to a high level of greenhouse gas capture, decreases landfill, and correlates with the idea of a circular economy. The commercialization potential of biomass-based supercapacitors is supported by recent developments in AI-based optimization, combined with scalable synthesis methods, which would support ecologically, economically, and technologically sustainable energy storage on a large scale.

## 1. Introduction

The global energy landscape is experiencing a significant transformation, primarily attributed to intensifying environmental challenges, increased population growth, and the continual rise in global energy demand. Currently, fossil fuels provide more than 80% of the global primary energy, which refers to finite and high-level sources of greenhouse gas production. As the global electricity consumption is expected to rise over 35,000 TWh by 2040, there is an imminent need to come up with long-term and viable energy sources that correspond with the decarbonization goals [1]. Renewable energy resources such as solar and wind energy are becoming the foundation of future energy systems; nevertheless, the intermittency and unpredictability of these renewable energy sources pose serious challenges related to the grid stability of providing a reliable energy supply.

**Figure 1:**
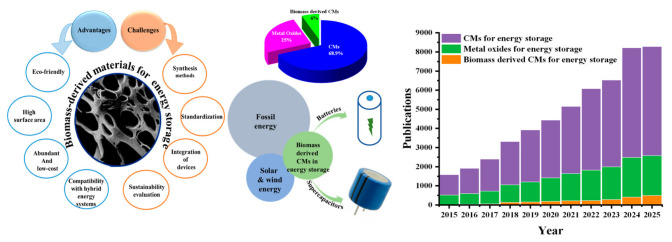
Global energy distribution shows the dominance of fossil fuels and the growing role of renewables. The contribution of biomass-derived CMs in energy storage, pie chart showing research distribution and bar chart is about the rising annual publications from 2015 to 2025 (data were taken from the ‘Scopus’ database on 26 October 2025 using the terms ‘CMs in energy strorage, metal oxides in energy storage, and biomass derived CMs in energy storage’ to search within article title, abstract, and keywords).

To guarantee energy sustainability in the world and allow a smooth shift to carbon neutrality, energy storage has been identified as a key solution [2]. As provided in Figure 1, the global energy structure remains dominated by fossil fuels, while renewable and biomass-derived materials are gaining increasing attention for sustainable energy storage. It is estimated that global installed energy storage capacity will exceed 400 GW by 2030, representing a fivefold increase compared to 2020. Large-scale storage technologies like pumped hydro and hydrogen systems are effective in long-term storage [3]. Alternatively, electrochemical storage provides a flexible, scalable storage with integration opportunities in both stationary and mobile applications. In some countries, such as India, it is implied that only with the addition of new advanced storage technologies that equalize seasonal differences and daily loading patterns can a 100% renewable electricity system become technically viable by 2050 [2]. Therefore, sustainable storage is not simply a technological choice but a necessity for achieving long-term energy security and climate targets.

Supercapacitors have attracted significant attention due to their unique electrochemical properties. Conventional rechargeable batteries, such as lithium-ion batteries, provide high energy densities (150–250 Wh kg^−1^) with inadequate power density and cycle life. By comparison, conventional capacitors have very high power density (>10 kW kg^−1^) and minimal energy storage. Electrochemical capacitors, also known as supercapacitors, provide an intermediate solution, with energy densities of 530 Wh kg^−1^ and power densities as high as 10 kW kg^−1^ and ultralong cycle life exceeding 100,000 charge–discharge cycles [4]. Developments like hybrid supercapacitors combine faradaic and capacitive charge storage systems, effectively combining the improvement of batteries and capacitors. These systems have demonstrated an energy density of 80 to 100 Wh kg^−1^, fast kinetics of charge and discharge, stability, and have been applied to electric vehicles, regenerative braking, and renewable energy buffering [5]. Dual redox-active ion electrolytes also lead to higher power–energy balancing, increasing the scope of supercapacitor to grid-level storage [4]. Supercapacitors are also gaining popularity in the current energy storage portfolio due to their high power, safety, and durability.

Electrode materials determine the performance of supercapacitors. Carbon-based structures prevail in terms of their conductivity, stability, and porosity that can be regulated. Synthetic carbons require significant energy input; in contrast, biomass-derived carbons are renewable, inexpensive, and green, and utilize enormous natural resources and streams of waste. Billions of tons of lignocellulosic biomass and agricultural residues are produced annually around the world, which provides numerous precursors with heteroatoms (N, O, S) capable of improving electrochemical activity [6]. The capabilities of biomass-derived carbons are demonstrated in recent studies. Xia et al. (2025) [7] reported the dual-salt-activation of the cellulose-chitosan biomass and obtained hierarchical porous carbons, characterized by surface areas of over 2000 m^2^g^−1^ and capacitances of over 350 F g^−1^. Furthermore, Teng et al. (2025) [8] established that the relative contributions of cellulose, hemicellulose, and lignin determine the pore structure and doping characteristics, resulting in carbons with capacitances exceeding 400 F g^−1^ with 95% retention at 10,000 cycles. These findings demonstrate how biomass valorization can be extended to the principles of the circular economy by converting waste into high-value electrode materials. The hierarchical porosity and heteroatom-enriched structure of biomass carbons facilitate ion-mass transport and pseudo-capacitance, permitting the high-rate operation and reduction in the carbon footprint relative to synthetic carbons [6].

Hierarchical biomass-derived materials with interconnected pore networks have been shown to deliver high specific capacitances of 450–500 F g^−1^ and energy densities exceeding 35 Wh kg^−1^ [9]. Moreover, Zhu et al. (2025) [10] reviewed the synthesis of biomass-derived carbon- and zinc-based supercapacitors, highlighting synergistic design strategies. In several reported systems, biomass-derived carbons combined with zinc electrodes achieved volumetric energy densities above 25 mWh cm^−3^ and areal capacitances of 5–10 F cm^−2^ under high-rate conditions.

Activated carbons derived from *Osmanthus fragrans* have demonstrated specific surface areas exceeding 2100 m^2^ g^−1^, with energy densities of 38 Wh kg^−1^ at power densities of 750 W kg^−1^ [11]. Similarly, systematic studies on biomass-derived carbons in sodium-ion systems have shown that optimized graphitization and pore structure significantly enhance electronic conductivity and capacity retention by more than 20% compared to conventional carbon materials [12].

Innovations such as dry electrode processing have further reduced the environmental impact of energy storage systems, increasing volumetric energy density by approximately 30% while eliminating the use of toxic solvents [13]. Moreover, the integration of supercapacitors with batteries has been shown to extend battery cycle life by 15–20% in electric vehicles, owing to the complementary energy storage characteristics of the two systems [14].

Life cycle assessment (LCA) studies further indicate that biomass-derived carbon electrodes can reduce the environmental footprint by up to 40% compared to graphene-based electrodes, highlighting their superior sustainability performance [15].

From a technical perspective, biomass valorization aligns with waste management and climate goals. Biomass waste, including agricultural residues and food waste, represents a potential feedstock of hundreds of millions of tons annually [16]. These waste streams have the potential to counter carbon emissions, alleviate landfill pressure and offer inexpensive electrode materials once they are converted into carbons. Biotechnological solutions, like the recovery of lactic acid by fermentation of microbes, are also facilitating the integrated waste-to-energy solutions [17]. Altogether, these findings demonstrate that biomass-derived carbons not only improve the performance of supercapacitors but also help to attain the global sustainability goals.

This review focuses on the potential of biomass-derived carbon materials for high-performance supercapacitor applications. Unlike previous review articles that primarily provide general discussions on biomass-derived carbons [6,16], the present work specifically emphasizes food waste-derived carbons as a distinct and relatively underexplored precursor class. The main focus is on biomass-derived carbon materials, covering precursor diversity, activation mechanisms, structural design, and doping strategies, along with a comparative evaluation against conventional electrode materials.

In addition, emerging directions such as AI-assisted materials design and scalable green synthesis are critically discussed, highlighting that biomass-derived carbons are not only technologically promising but also environmentally essential in the broader context of sustainable energy storage and carbon neutrality.

## 2. Fundamentals of Supercapacitors

Supercapacitors, also known as electrochemical capacitors, are advanced energy storage devices that bridge the gap between conventional capacitors and batteries by combining high power density with moderate energy density [18]. They operate through reversible charge accumulation at the electrode–electrolyte interface, enabling rapid charge–discharge cycles, typically within seconds, and long cycle lifetimes exceeding 10^5^ cycles [19]. In contrast to batteries, which rely on faradaic redox reactions occurring in the bulk of electrode materials, supercapacitors store energy either through electrostatic ion adsorption or fast surface redox processes [20].

Supercapacitors store energy through two primary mechanisms: electric double-layer capacitance (EDLC) and pseudocapacitance. EDLC-based materials, such as activated carbon and biomass-derived carbons, store energy via electrostatic ion adsorption at the electrode surface without charge transfer, resulting in specific capacitances typically in the range of 100–250 F g^−1^ [21]. In contrast, pseudocapacitive materials, including transition metal oxides and conducting polymers, rely on fast and reversible surface redox reactions, enabling higher capacitances of up to ~1000 F g^−1^ and energy densities of 10–50 Wh kg^−1^ [22]. The fundamental energy storage relationship, indicates that stored energy is directly proportional to both operating voltage and specific capacitance [23].

High power densities (10–15 kW kg^−1^) in supercapacitors arise from low internal resistance and high ionic mobility within the electrolyte, making them suitable for applications requiring rapid power delivery, such as regenerative braking and backup power systems [24]. The charge–discharge mechanism involves rapid ion adsorption and desorption at the electrode–electrolyte interface, with minimal structural degradation compared to batteries, which typically undergo significant structural changes during repeated cycling [25].

The most significant distinction between the supercapacitors and the traditional capacitors is the electrochemical interface. Conventional capacitors store energy through dielectric polarization and usually have capacitances in the range of microfarads, while supercapacitors have nanostructured porous electrodes which multiply the surface area (up to 2000 m^2^ g^−1^) and allow ion accumulation at the nanoscale [26]. Supercapacitors exhibit higher power density and cycling stability with lower energy density, 5–30 Wh kg^−1^, in comparison to batteries (100–250 Wh kg^−1^), on average [27]. Sustainability and the ability to be compatible with biomass-derived carbon materials further enhance their position as the next generation energy storage devices in clean and efficient power systems [28].

**Figure 2:**
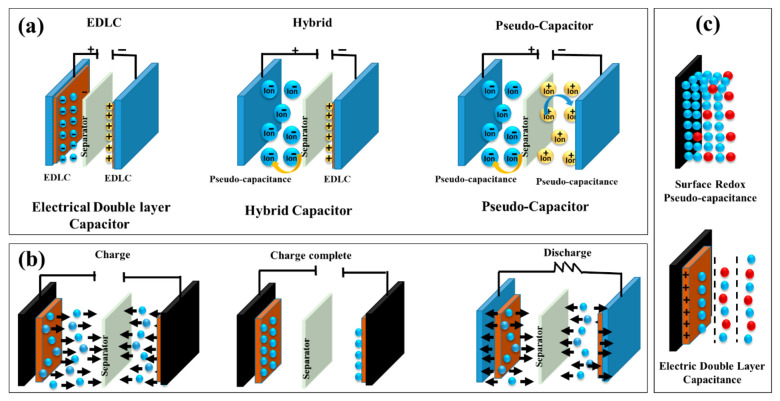
Different types and charge storage mechanisms of supercapacitors. (**a**) Classification of supercapacitors into electrical double-layer capacitor (EDLC), hybrid capacitor, and pseudocapacitor based on their energy storage principles. (**b**) Charging and discharging processes in EDLCs show adsorption and desorption at the electrode–electrolyte interface. (**c**) Comparison of electric double-layer capacitance and surface redox pseudocapacitance mechanisms.

Supercapacitors are generally classified into three major types: electric double-layer capacitors (EDLCs), pseudocapacitors, and hybrid supercapacitors based on their energy storage mechanisms and electrode materials [29]. As illustrated in Figure 2a, these three configurations differ in their charge storage mechanisms and electrode behaviour. EDLCs are used to store energy by the electrostatic separation of charges at the electrode/electrolyte interface without undergoing any faradaic reaction. The charge and discharge processes in EDLCs, shown in Figure 2b, occur through the reversible adsorption and desorption of ions at the electrode surface. The storage of energy in such systems is based on the adsorption of the electrolyte ions onto the surface of the high-surface-area carbon-based electrodes, including activated carbon, graphene, and carbon nanotubes [30]. The distinction between electric double-layer capacitance and surface redox pseudocapacitance is schematically represented in Figure 2c. These porous carbons have a large surface area (typically more than 2000 m^2^g^−1^), which allows high capacitance values of 100–250 F g^−1^ and high cycle stability of more than 10^6^ cycles [31]. The lack of chemical reactions guarantees high power densities and high operation lifetimes, which makes EDLCs promising in fast charge–discharge processes.

Pseudocapacitors store energy by means of fast redox reactions that are reversible and occur at or near the electrode surface in a fast faradaic reaction. Transition metal oxides (e.g., RuO_2_, MnO_2_, NiO, Co_3_O_4_) and conducting polymers (such as polyaniline and polypyrrole) are widely used for these devices [32]. However, their limited long-term stability compared to carbon-based EDLCs remains a key challenge. Due to their multi-electron transfer capabilities, these materials provide higher specific capacitances (SC) exceeding 500–1500 F g^−1^ and energy densities up to 50–80 Wh kg^−1^ [33]. Hybrid supercapacitors are a combination of EDLCs and pseudocapacitors, in which one of the electrodes is a non-faradaic one and the other a faradaic electrode [34], giving greater energy and power densities, usually reaching 30–80 Wh kg^−1^ and high-rate capacity with long cycle life [35]. Combining metal oxides or conducting polymers with carbon-based materials forms hybrid supercapacitors that fill the gap between batteries and traditional capacitors and are therefore of great potential in the next-generation sustainable and high-performance energy storage systems [36].

The charge storage processes in supercapacitors are mostly controlled by two different processes: the electric double-layer capacitance (EDLC) and the pseudocapacitance, which are essentially different in their energy storage modes and material properties [37]. Energy storage in EDLCs is achieved by adsorbing non-faradaic ions at the electrode electrolyte interface, and no charge transfer or chemical reaction occurs.

The charged ions of the opposite poles of the electrolyte become concentrated at the electrode surface when a potential is applied to establish a Helmholtz double layer that comprises an inner compact layer and an outer diffuse layer [38]. This is a highly reversible and fast process, so charge–discharge cycles can be performed at ultrafast rates, with capacitances of carbon electrodes usually ranging between 100 and 300 F g^−1^ graphene, carbon nanotubes and activated carbons [39]. This process relies on the accessibility of ions, pore structure, and surface area that may reach over 2000 m^2^ g^−1^ in optimized carbon materials [40].

Pseudocapacitance is a faradaic charge transfer reaction between redox, intercalation, or electrosorption surface/near-surface reactions [41]. In this case, the ions of the electrolyte are involved in fast and reversible oxidation-reduction reactions with the active electrode substance and result in greatly increased charge storage capacity. Pseudocapacitive behavior is observed in transition metal oxides like MnO_2_, Co_3_O_4_, and Nb_2_O_5_, and conducting polymers with capacitances of 500–1500 F g^−1^ and energy densities of up to 60–100 Wh kg^−1^ [42]. In contrast to EDLCs, pseudocapacitors can be ion-intercalated into the crystal structure, which allows transference of multiple electrons in addition to changes in volume with charging/discharging, which could compromise cycling stability [43].

Recent contributions to the 2D materials, e.g., MXenes and graphene-based nanocomposites, have made possible synergistic double-layer and pseudocapacitive behavior, with improved energy–power trade-offs [44]. These hybrid systems use both ion adsorption and redox reactions, effectively combining the high-power density of EDLCs with the high energy density of pseudocapacitors, and thus, they are very appropriate in sustainable and next-generation energy storage systems. To further clarify the differences between electric double-layer capacitance and pseudocapacitance. Table 1 provides a critical comparison of the mechanisms, materials, and electrochemical performance parameters of both systems, highlighting the synergistic capabilities of hybrid systems.

A combination of electrochemical parameters, including specific capacitance, energy density, power density, and cycling stability that determine the efficiency, durability, and applicability of supercapacitors, is critically dependent on the performance of supercapacitors in sustainable energy storage systems [48]. Table 2 provides a critical overview of the contribution of each metric to the total performance and practical optimization of the supercapacitors, a concise comparison of these essential parameters, their affecting factors, and relevant trade-offs.

Specific capacitance (F g^−1^) measures the amount of charge that is held by a unit mass of electrode material and is directly proportional to the electrochemical activity of the system. It is determined by the cyclic voltammetry (CV), galvanostatic charge–discharge (GCD), or electrochemical impedance spectroscopy (EIS) measurements [49]. Carbon-based EDLCs usually have particular capacitances of 100–250 F g^−1^, with pseudocapacitive or hybrid electrodes of 800–1500 F g^−1^ due to faradaic effects [50]. The capacitance and operating voltage obtain the energy density (Wh kg^−1^), which is the sum of stored energy. It is generally expressed as

(1) E=12CV2

where C is the capacitance, and V is the voltage window [51]. Traditional carbon-based EDLCs have a 5–15 Wh kg^−1^, and advanced hybrid and asymmetric designs may reach 40–80 Wh kg^−1^, comparable to the performance regime of batteries [52].

The rate of energy delivery is represented by power density (W kg^−1^) and is denoted

(2) $$P=\frac{E}{\Delta t}$$

where o is discharge time. EDLCs are usually capable of delivering 5–15 kW kg^−1^, and pseudocapacitors and hybrid supercapacitors demonstrate 2–10 kW kg^−1^ in different cases based on ion transport rates and electrode architecture [53]. High power density is especially needed where fast energy bursts are needed, e.g., regenerative braking and pulse power systems.

**Table 2:** The performance parameters of supercapacitors, their characteristics and the relationships among capacitance, energy density, power density, and long-term stability.

No	Parameter	Formula/Definition	Typical Range	Influencing Factors	Trade-Offs/Challenges	Ref.
1	Specific Capacitance (F g^−1^)	$C=\frac{I\Delta t}{m\Delta v}$; quantifies charge storage per unit mass	100–300 for carbon-based EDLCs; 500–1500 for pseudocapacitors	Electrode surface area, porosity, electrolyte type, and ion mobility	High capacitance in pseudocapacitors often leads to reduced cycle life and slower charge–discharge	[48,49,54,55]
2	Energy Density (Wh kg^−1^)	$E=\frac{1}{2}C{\left(\Delta V\right)}^{2}/3.6$	5–15 (EDLCs); 20–100 (pseudocapacitors); up to 150 (hybrid)	Capacitance, voltage window, and cell design	Increasing energy density may compromise power density and safety	[49,50,51,53]
3	Power Density (W kg^−1^)	$P=\frac{E}{\Delta t}\times 3600$	1000–10,000	Equivalent series resistance (ESR), ion diffusion, and electrode–electrolyte interface	High power density usually reduces energy density	[49,50,51]
4	Cycling Stability	Number of charge/discharge cycles with minimal performance loss	>100,000 for carbon-based; 10,000–50,000 for metal oxides and hybrids	Material structure, redox reversibility, and electrolyte stability	Metal oxides and polymers degrade faster due to volumetric changes during cycling	[48,56,57]
5	Retention (%)	Ratio of final to initial capacitance after repeated cycling	80–100% over 10^4^–10^5^ cycles	Structural robustness, conductive network integrity	Low retention limits long-term durability	[52,56,57]
6	Parameter Balance for Practical Applications	Optimization of C, E, P, and stability to meet real-world demands	Depends on application (EVs, portable devices, grid systems)	Electrode–electrolyte compatibility and device architecture	Need to balance high energy with fast kinetics and longevity	[50,52,57,58]

Cycling stability is a measurement of the long-term stability of supercapacitors. Typically, EDLCs maintain capacitance greater than 95% after 10^6^ cycles, compared to 80–90% maintenance by pseudocapacitors after 10^4^–10^5^ cycles, primarily because structural degradation takes place in the redox processes [56]. Biomass-derived carbons, optimized electrode structures, and hybrid structures are important to enhance retention and structural stability [57]. The balancing of these parameters to deliver high energy density without affecting power delivery and cycling stability is critical in practical applications. Supercapacitors can be developed with a high-power output and a long service life of the device by using advanced electrode materials and perfected device configurations, making them viable in their next-generation sustainable energy storage technologies [50].

## 3. Food Waste Biomass Derived-Carbon Materials

Food waste biomass represents the primary focus of this review, serving as a rich, inexpensive, and renewable resource to produce an energy-storing and environmentally applicable high-performance carbon material. To provide a structured framework, food waste biomass is systematically classified in this review into four primary categories: (i) fruit-derived wastes (e.g., peels and seeds), (ii) vegetable residues (e.g., stalks and skins), (iii) cereal-based wastes (e.g., rice husks and wheat bran), and (iv) oilseed by-products (e.g., press cakes and shells). Each category exhibits distinct biochemical composition and mineral content, which directly influence carbon yield, pore structure, heteroatom distribution, and electrochemical performance. Fruit-derived wastes such as orange, banana, and mango are the sources of lignocellulose, hemicellulose and essential oils [59]. After pyrolysis and activation, these wastes yield porous carbons with high specific surface areas exceeding 1500 m^2^ g^−1^ and specific capacitances of up to 310 F g^−1^, attributed to oxygen-containing functional groups that enhance ion transport and electrolyte wettability [60].

Vegetable residues, stalks, and skins contain fibrous cellulose and nitrogen-containing compounds, thereby making N-doped carbon structures. These doped carbons are more electrically conductive and have a high energy density, with 45–60 Wh kg^−1^ being the highest observed in supercapacitors [61]. High silica and carbon content are found in cereal-based wastes such as rice husks and wheat bran. Hydrothermal carbonization or chemical activation leads to their conversion to hierarchical porous structures with equal micropores and mesopores, allowing good ion accessibility and cycling stability over 10,000 cycles [62].

Oilseed by-products, including press cakes and soybean or sunflower shells, contain significant carbon content (~50 wt%) and residual oils, which can contribute to self-activation during carbonization. These materials typically exhibit specific surface areas in the range of 1200–1600 m^2^ g^−1^ and high energy densities of approximately 70 Wh kg^−1^, making them promising candidates for sustainable supercapacitor electrodes [63].

Biomass-derived carbons obtained through food waste valorization not only help reduce environmental burdens but also provide functional electrode materials with tunable morphology and favorable electrochemical properties. In addition, their inherent scalability makes them attractive for applications in green energy storage systems [64].

The biomass of food waste, mainly the chemical constituents of cellulose, hemicellulose, lignin, proteins, lipids and minerals, is a significant factor in determining the carbon yield, porosity, conductivity and heteroatom doping properties of the resulting carbon materials [65]. Cellulose and hemicellulose are polysaccharides rich in carbon and hydroxyl groups, which decompose in the range of 250–400 °C, releasing volatile gases and forming char residues. They typically constitute 50–70 wt% of food waste biomass [66]. They can undergo controlled thermal degradation, which facilitates the creation of microporous and mesoporous carbon structures, with surface areas commonly in excess of 1000 m^2^ g^−1^, that increase ion transport and electrochemical activity [67].

Lignin is a highly aromatic and thermally stable polymer that contributes to higher carbon yield and structural rigidity during carbonization, typically accounting for 10–30 wt% of biomass composition [68]. Its aromatic structure facilitates graphitization and improves electrical conductivity, leading to the formation of stable electrode materials with enhanced charge–discharge reversibility [69].

Proteins and lipids serve as natural sources of nitrogen, sulfur, and phosphorus dopants, introducing defects and active sites within the carbon framework. In particular, nitrogen doping enhances pseudocapacitive behaviour and electron transfer by increasing surface polarity and improving electrolyte wettability [70].

Minerals such as potassium, calcium, and magnesium salts act as inherent activating agents during pyrolysis, promoting pore formation and surface roughness development [71]. These inorganic components contribute to self-activation, leading to the formation of hierarchically porous carbons with enhanced electrochemical activity. Collectively, these chemical constituents control not only the textural and electrical properties but also the heteroatom distribution within the carbon structure, making food waste biomass a multifunctional precursor for high-performance energy storage materials [72].

A critical summary of the results concerning the effect of individual biochemical components on the physicochemical and electrochemical behaviour of the resultant carbons is provided in Table 3.

Biomass precursors derived from food waste are strongly influenced by their chemical composition and heteroatom content, which significantly affect the structure and electrochemical properties of the resulting carbon materials. This relationship between precursor chemistry and carbon structure is reflected in surface area, pore size distribution, and electrical conductivity, which directly determine charge storage capability [77].

Hemicellulose- and cellulose-rich precursors typically require longer processing times to yield carbons with high specific surface areas (1000–2000 m^2^ g^−1^) and well-developed microporosity, which facilitates ion adsorption and electric double-layer formation [78]. In contrast, lignin- and protein-rich biomasses tend to produce more graphitic carbons with higher electrical conductivity and increased mesoporosity, which enhance ion diffusion kinetics and reduce internal resistance during electrochemical cycling [79].

The inherent natural doping of food biomass with nitrogen (N), sulfur (S), phosphorus (P), and oxygen (O) atoms is a major way to enhance electrochemical performance by providing extra active sites and altering surface polarity [80]. Pseudocapacitive behaviour and charge transfer are improved through nitrogen doping, particularly with pyridinic and graphitic N sites, while oxygen-containing functional groups enhance wettability and increase ion accessibility. Sulfur and phosphorus atoms, derived from amino acids, phospholipids, and proteins, provide electron-donating centres and redox-active sites, thereby enhancing specific capacitance and energy density [81]. For instance, N/O co-doped carbons produced from fruit and vegetable residues exhibit specific capacitances of up to 350 F g^−1^ and cycling retention greater than 95% after 10,000 cycles [82].

It determines that the hierarchical pore architecture and surface chemistry of the resultant carbons are determined by the synergetic interaction of precursor composition, activation conditions, and heteroatom self-doping, resulting in improved ion transport, high conductivity, and better electrochemical energy storage performance.

### 3.1. Synthesis and Characterization

**Figure 3:**
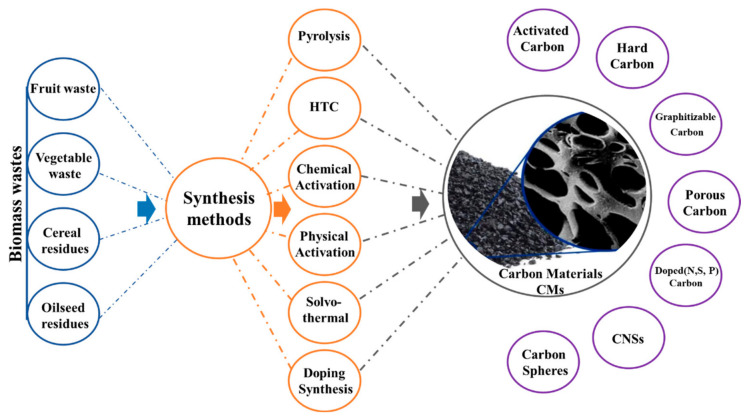
Food waste biomass sources, synthesis methods and different types of carbon materials for supercapacitor applications.

The synthesis of biomass-derived carbon materials (BDCMs) is critical for tailoring their physicochemical properties, such as porosity, morphology and surface chemistry. Figure 3 shows that different types of biomass waste, like fruit residues, vegetable peels, cereal husks, and oilseed by-products, are commonly used as renewable carbon sources because of their abundance and natural heteroatom composition [83]. These precursors have many synthesis pathways, such as pyrolysis, hydrothermal carbonization (HTC), chemical and physical activation, solvothermal treatment, and doping synthesis. Every method has an individual effect on the carbon structure, graphitization extent, and electrochemical performance [84].

Pyrolysis is the most conventional and scalable approach, involving thermal decomposition of biomass under inert or limited-oxygen atmospheres to generate amorphous or partially graphitic carbons. The activation step, either chemical (KOH, ZnCl_2_, or H_3_PO_4_) or physical (CO_2_ or steam), further enhances a hierarchical pore structure which is essential for rapid ion transport and high capacitance [85]. Chemical activation is crucial in developing hierarchical porosity. During activation, reagents (KOH, ZnCl_2_, or H_3_PO_4_) react with the carbon matrix and promote pore formation through redox reactions, dehydration, and gas evolution processes. The intercalation of potassium species into the carbon lattice expands the structure, while the release of gaseous products generates abundant micropores and mesopores. Furthermore, H_3_PO_4_ promotes cross-linking and dehydration reactions that stabilize the carbon framework [85].

In contrast, hydrothermal carbonization (HTC) enables carbon formation at relatively low temperatures (180–250 °C) in aqueous media, producing oxygen-rich hydrochars with tunable morphology and abundant surface functional groups, which can subsequently be activated or doped to enhance electrical conductivity [83]. Solvothermal and doping strategies introduce heteroatoms (N, S, P, and O) into the carbon structure, improving wettability and redox activity through defect engineering [83].

As illustrated in Figure 3, these synthesis approaches yield a wide range of carbon materials, including activated carbons, hard and graphitizable carbons, porous carbons, carbon spheres, carbon nanosheets (CNSs), and heteroatom-doped carbons, each offering specific advantages for energy storage applications [86]. The morphology and pore architecture established during synthesis strongly influence ion accessibility, electrical conductivity, and electrochemical kinetics. Therefore, the selection of biomass precursor and synthesis pathway is critical for optimizing energy storage performance, highlighting how sustainable carbon production can be integrated with high-performance electrochemical functionality [84].

Another important aspect in the study of biomass-derived carbon materials is their characterization, which is essential for evaluating their physicochemical, structural, and electrochemical properties. These materials are typically analyzed using a combination of complementary techniques to obtain a comprehensive understanding of their properties. Scanning electron microscopy (SEM) and transmission electron microscopy (TEM) provide detailed morphological information, including surface texture, pore structure, and particle distribution, which are crucial for understanding the formation mechanisms and connectivity of carbon frameworks [87]. The crystal structure and purity of the phase are studied with X-ray diffraction (XRD), and the functional groups, bonding forms, and the level of graphitization are analyzed with the assistance of the Fourier-Transform infrared (FTIR) and Raman spectroscopy [88].

The Brunauer–Emmett–Teller (BET) technique is used to determine specific surface area, pore size distribution, and total pore volume, which are critical parameters for evaluating ion diffusion and adsorption [89]. Elemental composition and chemical states are analyzed using energy-dispersive X-ray spectroscopy (EDX) and X-ray photoelectron spectroscopy (XPS) to identify heteroatom doping and surface functionalities [90]. Electrochemical characterization techniques, including cyclic voltammetry (CV), galvanostatic charge–discharge (GCD), and electrochemical impedance spectroscopy (EIS), are employed to evaluate charge storage mechanisms, electrical conductivity, and cycling stability of the electrode materials [91].

The integration of these complementary techniques provides a comprehensive understanding of structure–property relationships in biomass-derived carbons, which is essential for optimizing their electrochemical performance in sustainable energy storage systems.

**Figure 4:**
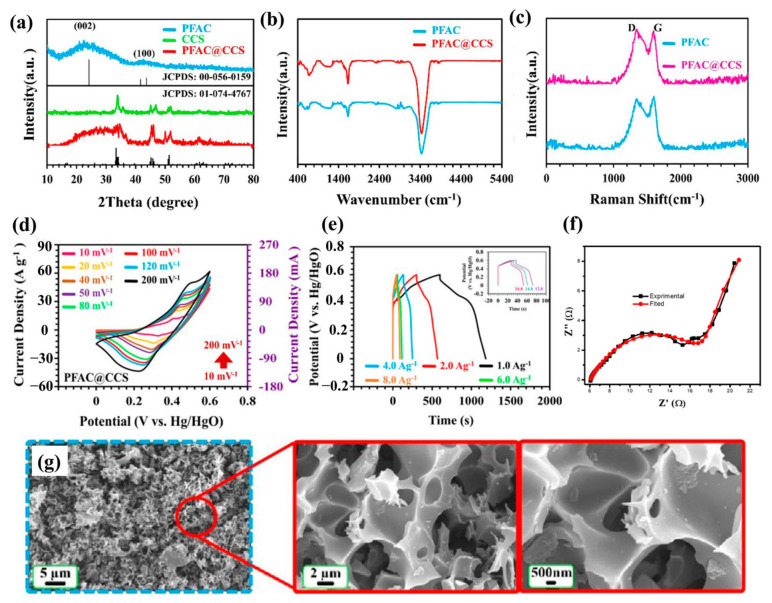
(**a**) XRD; (**b**) FTIR; (**c**) Raman; (**d**) Cv; (**e**) GCD; (**g**) SEM analysis of PFAC and PFAC@CCS and (**f**) EIS of activated carbon derived from Hyparrhenia hirta. [(**a**–**e**,**g**) figures were reproduced from Tavakoli, 2025 [92]; and figure from Duraisamy, 2026 [93] which is published under CC-By 4.0].

Figure 4a–e,g present the systematic characterization of pristine porous food-waste activated carbon (PFAC) and the PFAC@CCS composite, while Figure 4f shows electrochemical impedance spectroscopy (EIS) results for activated carbons (ACs) derived from *Hyparrhenia hirta* biomass. Collectively, these results provide comprehensive insights into the structural, spectroscopic, electrochemical, and morphological properties of the materials.

The XRD patterns (Figure 4a) exhibit broad diffraction peaks at approximately 24° and 43°, corresponding to the (002) and (100) planes of amorphous carbon with turbostratic graphitic domains. In the PFAC@CCS composite, additional crystalline reflections are observed, which are attributed to the embedded CuCo_2_Se_4_ phase, confirming successful integration of the active component. A slight improvement in graphitic ordering is also evident. This increase in structural ordering enhances electronic conductivity and charge transport, consistent with previous reports on biomass-derived hybrid carbon materials [92].

The FTIR spectra in Figure 4b confirm the presence of oxygen-containing surface functional groups, evidenced by characteristic stretching vibrations at 3420 cm^−1^ (–OH), 1620 cm^−1^ (C=O), and 1100 cm^−1^ (C–O–C). These functional groups enhance electrolyte wettability and ion accessibility, which are essential for pseudocapacitive behaviour. A noticeable decrease in the intensity of these peaks is observed in the PFAC@CCS composite, suggesting partial removal or transformation of oxygen functionalities during the compositing process. This modification results in a more conductive surface with reduced defect density, consistent with previous reports on chemically activated biomass-derived carbons and transition-metal/carbon composites [94].

The Raman spectra in Figure 4c display two prominent bands: the D band (~1350 cm^−1^), associated with structural disorder, and the G band (~1580 cm^−1^), corresponding to graphitic sp^2^-hybridized carbon. The PFAC@CCS composite exhibits a lower ID/IG ratio compared to pristine PFAC, indicating a higher degree of graphitization and reduced structural disorder. This enhanced structural ordering is attributed to the catalytic effect of the CCS phase during pyrolysis, which promotes the growth of graphitic domains and improves electrical conductivity. Similar Raman features have been reported in heteroatom-doped and metal oxide/carbon hybrid materials derived from renewable biomass sources [95].

These structural improvements are further supported by electrochemical characterization. The cyclic voltammetry (CV) curves of PFAC@CCS (Figure 4d) exhibit quasi-rectangular shapes with weak redox humps, indicating the coexistence of electric double-layer capacitance (EDLC) and pseudocapacitance. The increase in CV loop area with increasing scan rate demonstrates excellent rate capability and rapid ion diffusion, attributed to the interconnected porous network and conductive carbon framework.

The galvanostatic charge–discharge (GCD) curves display nearly symmetric profiles across different current densities, indicating high coulombic efficiency and good electrochemical reversibility (Figure 4e). PFAC@CCS exhibits higher specific capacitance and more stable charge–discharge behaviour compared to pristine PFAC, consistent with other metal–carbon composites that benefit from enhanced redox activity and electrical conductivity [92].

The EIS analysis in Figure 4f shows a Nyquist plot with a small semicircle at high frequency and a nearly vertical line at low frequency. The low intercept indicates minimal equivalent series resistance (R_s), reflecting good electrical conductivity [93]. The small semicircle suggests low charge transfer resistance (R_ct), confirming efficient electron transport at the electrode–electrolyte interface. The steep low-frequency line indicates ideal capacitive behavior and rapid ion diffusion. This performance is attributed to the hierarchical meso/microporous structure of the activated carbon derived from Hyparrhenia hirta, enabling efficient ion transport and superior electrochemical performance for supercapacitor applications.

The SEM images in Figure 4g reveal that PFAC@CCS exhibits a porous, interconnected network structure with hierarchical micro- and mesopores. These pores facilitate electrolyte penetration, shorten ion transport pathways, and increase the electrochemically active surface area, all of which are critical for achieving high power and energy density. CCS nanoparticles are uniformly distributed throughout the carbon framework, forming a stable and continuous interface that enhances mechanical strength and electrical conductivity.

Similar porous graphitic structures have also been reported in biomass-derived carbons obtained from fruit and tea leaf precursors, which demonstrate efficient ion transport and long-term stability in supercapacitor applications [96]. Overall, these findings indicate that PFAC@CCS possesses improved graphitic ordering, optimized porosity, and enhanced electrochemical performance, attributed to the synergistic interaction between PFAC and CCS, making it a promising electrode material for high-performance supercapacitors [92].

### 3.2. Environmental and Economic Aspects

The ecological and economic nature of biomass-derived carbon materials (BDCMs) when used in supercapacitor applications constitutes one of the pillars of sustainable energy studies. The combination of the principles of a circular economy and waste-to-resource solutions converts biomass residues, which are agricultural, lignocellulosic, and food wastes, into high-value carbon substances, which in turn decrease the amount of waste and ensure renewable cycles of material. Compared to traditional synthetic carbon feedstocks (e.g., petroleum coke or graphene oxide), biomass feedstocks are abundant, low-cost, and renewable, offering both environmental and economic benefits. The hydrothermal and pyrolytic conversion of food and lignocellulosic residues into carbon-based materials reduces landfill burden and decreases methane and CO_2_ emissions typically associated with biomass decomposition [97].

The circular economy model is based on the concept of waste valorization through the recovery of energy and materials from discarded biomass, thereby establishing a closed loop between production, consumption, and disposal. Such a transition supports the achievement of global carbon neutrality and reduces reliance on non-renewable resources. Studies on hydrothermal carbonization of food waste have demonstrated that circular valorization can reduce greenhouse gas (GHG) emissions by approximately 60–70% compared to landfill or incineration pathways [98]. Furthermore, lignocellulosic-derived activated carbons exhibit a lower life-cycle carbon footprint and reduced energy demand during activation compared to fossil-derived carbons, while maintaining comparable or higher specific capacitance values exceeding 250 F g^−1^ [99]. These findings highlight the dual advantages of environmental sustainability and high electrochemical performance. As shown in Table 4, food waste, lignocellulosic residues, and agri-food by-products represent diverse biomass sources that offer significant reductions in GHG emissions and production costs, alongside high electrochemical efficiency.

Comparative data give a clear picture of how waste-to-resource valorization can improve environmental performance while also ensuring economic viability, thereby supporting the practical implementation of the circular economy framework in the development of sustainable supercapacitors.

The economic feasibility of biomass-based carbon synthesis lies in the reduction in raw material and processing costs. Agricultural wastes such as tea residues, coconut shells, and rice husks are generally low-cost feedstocks. However, practical implementation may involve additional expenses related to collection, transportation, and preprocessing, although these costs remain significantly lower than those of conventional synthetic precursors. Economic analyses show that biomass-derived activated carbons can be produced at 40–60% lower cost than those synthesized from synthetic precursors due to lower energy requirements and decentralized processing systems [100]. Tea waste-derived porous carbons, for example, exhibit high surface area (≈950 m^2^ g^−1^) and competitive energy densities (~38 Wh kg^−1^), validating their cost-to-performance ratio in scalable production [105].

From an environmental standpoint, biochar and hydrochar formation pathways contribute significantly to carbon sequestration and GHG mitigation. The carbonization process stabilizes organic carbon, preventing its release as CO_2_ or CH_4_, while simultaneously producing porous carbon for energy devices [102]. The valorization of lignocellulosic and food waste not only reduces landfill accumulation but also offsets fossil carbon dependency, thus aligning with United Nations Sustainable Development Goals [101]. The conversion of one ton of biomass waste into activated carbon could reduce over 1.5 tons of CO_2_-equivalent emissions through both sequestration and substitution effects [103].

The environmental and economic performance of biomass-derived carbon materials demonstrates a synergistic sustainability advantage. By integrating waste management with clean energy production, these materials represent a practical implementation of circular economy principles within the energy sector. The waste-to-resource approach not only improves environmental quality through emissions reduction and resource recovery but also provides a cost-effective route for fabricating high-performance supercapacitor electrodes. Biomass-derived carbon materials constitute a key platform for bridging ecological responsibility and energy efficiency, offering a viable pathway toward sustainable, low-carbon, and economically feasible energy storage systems.

## 4. Biomass-Derived Carbon Materials for Supercapacitors

Food waste represents one of the most abundant and renewable biomass resources for the sustainable synthesis of carbon materials, offering a dual benefit of waste valorization and environmental protection. Globally, over 1.3 billion tons of food waste are generated annually, containing rich carbonaceous precursors such as cellulose, hemicellulose, lignin, starch, and lipids that can be thermochemically converted into porous carbon materials suitable for supercapacitor electrodes [16]. In contrast to metal, metal oxide, clay, and composite-based electrode materials, which, although capable of achieving high specific capacitances exceeding 600–1200 F g^−1^, often demand expensive precursors, complex synthesis routes, and suffer from limited cyclic stability [106,107,108].

Biomass-derived carbon materials offer a sustainable, low-cost, and environmentally benign alternative. Their renewable origin, hierarchical porosity, and heteroatom-doping potential enable comparable or even superior electrochemical stability and rate capability, positioning them as a promising green substitute for inorganic and composite materials in next-generation supercapacitors [109]. A comparative analysis of biomass-derived carbon materials with conventional electrode materials, including metals, metal oxides, clays, and composites, is presented in Table 5. This table highlights the differences in electrochemical performance, sustainability, and practical applicability, emphasizing that biomass-derived carbons combine environmental compatibility with competitive energy storage properties. The comparison further underlines their growing importance as sustainable alternatives in next-generation supercapacitor technologies.

Food waste can be broadly classified into four main categories: fruit wastes, vegetable wastes, cereal residues, and oilseed residues, each exhibiting distinct physicochemical properties that influence carbon yield, surface morphology, and electrochemical performance. Fruit wastes (e.g., peels and seeds) are typically carbon-rich and contain naturally occurring inorganic species such as potassium, calcium, and magnesium, which act as in situ activating agents during carbonization and enhance pore development. For example, banana and orange peels yield activated carbons with surface areas exceeding 1200 m^2^ g^−1^ and specific capacitances of up to 250 F g^−1^, which are attributed to their fibrous structure and intrinsic heteroatom content [73].

Fruit-based biomass is converted through CO_2_ activation or chemical processing to microporous carbons that can be used in high rates of ion movement in supercapacitors. Fruit waste-derived carbon nanodots have high photoluminescence and probable multifunctional energy conversion capabilities [113]. Lignocellulosic material is also obtained very well from vegetable wastes (stalks, skins). Such wastes like potato peels and cabbage stalks have been transformed into carbon structures that have moderate surface areas (600–900 m^2^ g^−1^) and energy densities of up to 20 Wh kg^−1^ [114]. These nitrogen and oxygen functionalities of the plant proteins and polysaccharides enhance the wettability of the electrode and pseudocapacitance behaviour, which increases the capacity to store charge [115].

Another feedstock that is widely available, particularly in developing regions, is cereal residues (husks, bran). Rice and wheat husks contain high amounts of silica and carbon, which are highly beneficial for producing hierarchical porous carbons. Carbons derived from wheat husks have exhibited surface areas exceeding 1400 m^2^ g^−1^ and specific capacitances of approximately 300 F g^−1^, demonstrating excellent energy storage potential [110]. Oilseed residues (press cakes, shells), such as soybean and groundnut cakes, contain high amounts of fixed carbon and low ash content. Their pyrolysis yields carbons with well-developed pore networks and surface areas of up to 1000 m^2^ g^−1^, making them suitable for electrode fabrication [116]. Nitrogen doping is also facilitated by the intrinsic lipid and protein structures, enhancing the electrical conductivity and electrochemical stability of the resulting carbon materials.

### 4.1. Electrochemical Performance

Biomass-derived porous carbon materials have emerged as promising electrode candidates for high-performance supercapacitors due to their tunable porosity, high specific surface area, and abundant heteroatom functionalities. Activation and templating processes convert food residues into hierarchical porous carbons (HPCs) comprising interconnected micropores (<2 nm), mesopores (2–50 nm), and macropores (>50 nm), which collectively enhance ion transport and charge storage. Micropores facilitate electric double-layer formation, mesopores enable rapid ion diffusion, and macropores act as ion-buffering reservoirs, improving electrolyte accessibility and reducing internal resistance [117].

The development of these porous architectures is strongly dependent on the activation strategy, including physical activation (e.g., CO_2_ or steam) and chemical activation with agents such as KOH, ZnCl_2_, or H_3_PO_4_, which govern pore distribution and the evolution of carbon structure. In particular, chemical activation with KOH typically produces a high density of micropores and specific surface areas exceeding 2000 m^2^ g^−1^, whereas physical activation allows better control over meso- and macropore formation and structural stability [118,119]. The integration of optimized activation routes enables the formation of hierarchical ion-transport channels, thereby enhancing ionic conductivity and cycling stability, which are critical for high-power applications [120].

In addition, heteroatomic doping and functionalization of surfaces also improve the electrochemical response of food waste carbon. In the carbonization process, inherent components like nitrogen, oxygen, phosphorus, and sulfur are also not lost or deliberately added to form redox-active sites and defect-rich regions and enhance wettability and pseudocapacitance [121]. Nitrogen doping enhances the electronic conductivity and leads to other faradaic reactions, whereas oxygen-based or phosphorus-based groups enhance ion diffusion and interfacial charge transfers [122]. These structural and chemical modifications provide outstanding electrochemical performance, and particular capacitances frequently surpass 300 F g^−1^ and energy densities of 45 Wh kg^−1^. The pore structures that are formed by the carbonization of food waste, hence, represent an efficient, sustainable path in generating next-generation supercapacitor electrodes, connecting waste upgrading with the development of new material structures to store renewable energy.

Surface functionalization and heteroatom modification of porous carbon materials made of food waste improve the electrochemical performance of the materials significantly by regulating the physicochemical and electronic properties of the carbon skeleton. The addition of nitrogen (N), oxygen (O), sulfur (S) and phosphorus (P) atoms creates plenty of active sites and changes the local electronic density, enhancing wettability and conductivity. These heteroatoms facilitate redox reactions, accessibility of electrolyte ions and increase the rate of charge transfer at the electrode–electrolyte interface [123]. N-doping in particular leads to the enhancement of the electron mobility under graphitic-N and pyridinic-N structures, whereas O- and P-doping introduce oxygenated and phosphate functional groups that enhance pseudocapacitive activity and hydrophilicity [124].

S-doping in turn distorts the electronic structure, which creates defect sites that increase the rate of ion diffusion and faradaic interactions [125]. The level of synergies is provided by co-doped systems, i.e., N/S, N/P, or multi-heteroatom frameworks, which increase charge storage capacities and raise cycling durability [126]. Biomass-derived porous carbons and doped materials have been summarized in Table 6, which indicates both quantitative and qualitative findings of recent literature.

As demonstrated in Table 6, hierarchical porosity (micro-, meso-, and macropores) and surface heteroatom doping have a combined beneficial effect on specific capacitance, conductivity, and cycling stability. It further shows that the synergistic impacts of co-doping and pore engineering are mostly to enhance the rate of ion diffusion and electrochemical exploitation of the active surface area, and this further supports the above discussion. However, the precise spatial distribution of heteroatoms within the carbon matrix and their interaction mechanisms are not yet fully understood due to limitations in advanced characterization techniques. Establishing a direct correlation between heteroatom distribution, synergistic effects, and electrochemical performance remains an open research challenge requiring further systematic investigation.

In addition to heteroatom doping, metal oxide modification further enhances the pseudocapacitive response. Incorporation of redox-active oxides such as NiO, MnO_2_, and Co_3_O_4_ onto biomass-derived carbon scaffolds enables reversible surface redox reactions, thereby improving specific capacitance and rate performance. The carbon support provides a conductive matrix that accommodates mechanical strain during cycling and facilitates rapid electron transport. As a result, such hybrid composites typically exhibit capacitances exceeding 1000 F g^−1^ [130]. It is also observed that emerging conductive polymer (polyaniline, Poly(3,4-ethylenedioxythiophene) (PEDOT)) and MXene (Ti_3_C_2_T_x_) hybrid architectures have excellent synergistic behaviour. Conductive polymers enhance flexibility and pseudocapacitance with faradaic charge storage, and MXenes enhance conductivity and interfacial charge mobility, creating stable hybrid electrodes with high energy density and fast charge–discharge kinetics [125].

One of the most promising directions is the use of metal–organic frameworks (MOFs) and their derivatives. Conductive MOFs, such as Cu_3_(HHTP)_2_ (where HHTP denotes 2,3,6,7,10,11-hexahydroxytriphenylene), exhibit intrinsic electrical conductivity and well-defined porosity, whereas MOF-derived carbons, sulfides, and oxides offer high surface area and abundant tunable active sites. Ni-MOF, NiCo-MOF, and Co-MOF-derived sulfide composites have demonstrated superior electrochemical performance, with capacitances of 2150 F g^−1^, 2000 F g^−1^, and more than 2600 F g^−1^, respectively [124].

In addition, MOF/graphene, MOF/MXene, and MOF/polymer hybrids combine the structural versatility of MOFs with highly conductive supports, resulting in materials that enable fast ion diffusion, enhanced redox activity, and excellent long-term stability. These hybridization and functionalization strategies transform biomass-derived carbons from low-value porous structures into high-performance multifunctional electrode materials, bridging the gap between sustainable synthesis and high electrochemical performance in next-generation supercapacitors.

Significant comparisons are made between food waste-based carbons and the traditional carbon material, including activated carbon, graphene and carbon nanotubes (CNTs) and denote the balance between sustainability, cost-effectiveness and electrochemical performance. However, meaningful comparison requires standardized testing conditions, including identical electrolytes, voltage windows, electrode configurations, and mass loading, as variations in these parameters can significantly influence the reported electrochemical performance.

Carbons derived from food waste exhibit high specific surface areas (1200–2800 m^2^ g^−1^) and hierarchical porosity, providing efficient ion accessibility and rapid charge transport comparable to commercial activated carbon (800–2500 m^2^ g^−1^), but at significantly lower cost [132].

In addition, these materials are intrinsically heteroatom-doped (N, O, S, and P) due to the natural composition of the precursors, which enhances pseudocapacitive behaviour. As a result, they deliver specific capacitances of 350–520 F g^−1^ and energy densities exceeding 45 Wh kg^−1^ [133].

As shown in Table 7, food waste-derived carbons exhibit surface areas and electrochemical performance comparable to advanced carbon materials such as graphene, while offering superior sustainability, lower cost, and excellent cycling stability. These features make them promising candidates for scalable energy storage applications.

In contrast, graphene and carbon nanotubes (CNTs) exhibit higher electrical conductivity (~10^4^ S m^−1^) and superior mechanical stability; however, their high cost and the complexity of large-scale assembly limit practical applications. Food waste-derived carbons possess lower conductivity (~10^2^–10^3^ S m^−1^), which is compensated by abundant oxygen- and nitrogen-containing functional groups that enhance wettability and redox activity, enabling cycling stability of up to 95% after 10,000 cycles [137].

In addition, food waste-derived carbons demonstrate improved ion diffusion kinetics compared to conventional activated carbons, owing to their tunable meso-/microporous structures and surface functionalities that facilitate stronger electrolyte interaction [139].

Therefore, while graphene and CNTs are primarily suited for high-end applications, food waste-derived carbons represent cost-effective, high-performance, and sustainable alternatives, effectively bridging the gap between conventional activated carbons and advanced nanocarbons in next-generation supercapacitors [138].

The performance of food waste-derived carbons in supercapacitors shows a strong correlation between precursor chemistry, resulting structure, and electrochemical behavior, particularly in terms of capacitance, rate capability, and long-term cycling stability. During carbonization and activation, the development of surface functionalities and pore structures is governed by the intrinsic heteroatom content and organic composition of the biomass, which is typically rich in proteins, cellulose, and lignin, ultimately determining the electrochemical response [140].

For example, protein-rich precursors such as eggshell membranes and soybean waste yield nitrogen-doped carbons with enhanced pseudocapacitive contributions, whereas fruit peel-derived carbons contain abundant oxygen functional groups that improve surface wettability and ion adsorption [141].

The hierarchical micro-/mesoporous structure of these carbons facilitates efficient electrolyte penetration and rapid ion diffusion, which are key characteristics for electric double-layer capacitors (EDLCs). Carbons obtained via hydrothermal carbonization followed by KOH activation have demonstrated specific capacitances of 320–450 F g^−1^ and energy densities up to 48 Wh kg^−1^ in EDLC configurations [142].

In pseudocapacitive systems, charge storage is further enhanced by Faradaic reactions associated with surface heteroatoms and redox-active functional groups, resulting in capacitances of up to ~500 F g^−1^ with excellent rate performance even at high current densities [143]. Moreover, hybrid supercapacitors incorporating metal oxides such as MnO_2_ or Co_3_O_4_ exhibit synergistic effects, achieving energy densities of 60–75 Wh kg^−1^ while maintaining 90–95% capacitance retention after 10,000 cycles [89].

The performance evaluation approaches, such as cyclic voltammetry (CV), galvanostatic charge–discharge (GCD), and electrochemical impedance spectroscopy (EIS), have confirmed that optimized pore structure and surface chemistry are closely related to ion transport resistance and charge storage efficiency [144]. Altogether, systematic optimization of precursor type and synthesis parameters allows food waste-derived carbons to acquire balanced double-layer and pseudocapacitive properties, positioning them as versatile, sustainable, and high-performance electrode materials in next-generation EDLCs, pseudocapacitors and hybrid supercapacitors [89]. In addition to intrinsic material properties, electrode-level parameters such as mass loading and electrode thickness play a critical role in determining practical supercapacitor performance. Many studies report high specific capacitance at relatively low mass loadings (<1–2 mg cm^−2^); however, increasing mass loading or electrode thickness often leads to reduced ion accessibility, increased internal resistance, and limited electrolyte penetration. Consequently, this results in decreased rate capability and lower effective capacitance under realistic operating conditions.

Accordingly, these findings demonstrate that precise control over carbonization and activation can provide successful balancing of ion accessibility and electrical conductivity to produce high capacitive behavior. In particular, when natural heteroatoms of biomass precursors are integrated, both double-layer and faradaic-charge storing capabilities are obtained, enabling these materials to compete or even outcompete many traditional carbons. This leads to the possibility of food waste-produced carbons that can offer cost-effective and sustainable energy sources with high energy and power density, long-cycle life, and tunable electrochemical activity across a wide range of supercapacitor designs.

### 4.2. Challenges and Future Perspectives

Supercapacitors with biomass-derived materials on a large-scale basis are associated with multifaceted issues that include material synthesis, standardization of the process, integration of devices and sustainability evaluation. Even though laboratory-scale performance has shown high energy and power densities, there is no industrial translation yet because of variances in feedstock composition, energy-consuming activation strategies, and insufficient scalability of the synthesis processes [145]. Scalability and industrial practicability are especially limited by the variability of batches of biomass precursors and the challenge of ensuring homogeneity of pore structure and heteroatom doping on a large scale. Another critical limitation is the inherent difficulty in regulating the morphology and microstructure of bio-derived carbons, as the carbonization process largely preserves the intrinsic architecture of the precursor. In most cases, this results in predominantly amorphous carbon frameworks with limited graphitic ordering, which restricts precise tuning of electrical conductivity and ion transport pathways. This lack of structural control remains a key challenge for achieving consistent and high-performance electrode materials at scale. Traditional activation techniques based on the use of KOH, ZnCl_2_, or H_3_PO_4_ typically provide good surface areas (>2000 m^2^g^−1^) but are ecologically costly and costly to commercialize. Switching into green activation with CO_2_, steam, or bio-based activating agents, with hydrothermal carbonization or microwave-assisted processes is a plausible path to a more sustainable future and reduced energy consumption [146].

A major limitation is the lack of standardization in food waste processing, which directly affects the electrochemical consistency of biomass-derived carbons. Carbon yield, porosity, and surface chemistry vary significantly due to differences in precursor origin, moisture content, and mineral composition. The implementation of standardized preprocessing protocols, including drying, grinding, and controlled pyrolysis conditions, as well as unified reporting metrics such as BET surface area, pore size distribution, and mass loading, would significantly improve the reproducibility and comparability of both laboratory-scale studies and industrial-scale production [145].

At the industrial level, this variability can be mitigated through feedstock blending, compositional pre-screening, and real-time process control strategies to normalize precursor properties across different batches. Additionally, implementing standardized preprocessing protocols and adaptive carbonization conditions (e.g., temperature programming based on proximate/ultimate analysis) can significantly reduce batch-to-batch inconsistencies and ensure more uniform material performance.

Future possibilities include the incorporation of biomass-based carbons into hybrid energy storage systems, such as asymmetric supercapacitors, battery-supercapacitor hybrids, and solar-powered systems, which are developing fields. These hybrid systems are a combination of the mechanisms of double-layers and faradaic-charge storing capabilities to realize a high-power density and energy density. Nevertheless, the interfacial compatibility of the carbon and pseudocapacitive materials, the mechanical stability of cycling, and the scalability of the electrodes have not been addressed yet. Future research must focus on maximizing the electrode structure, binderless design, and fully covered surface to provide industrial flexibility [147].

The development landscape is being reshaped by emerging trends such as AI-assisted materials design and circular economy concepts. Machine learning and ensemble prediction models are increasingly capable of accurately mapping relationships between precursors, synthesis parameters, and electrochemical performance, thereby reducing experimental workload and associated costs [148]. In parallel, circular economy frameworks integrating waste collection, carbonization, and electrode fabrication within localized systems can enhance sustainability and strengthen supply-chain resilience [149].

Despite these advances, several critical gaps remain. Techno-economic and life-cycle assessments evaluating industrial feasibility are still limited. In addition, policies and infrastructure for large-scale food waste collection remain insufficiently developed, and scalable roll-to-roll electrode manufacturing has yet to be demonstrated. Addressing these challenges requires cross-sector collaboration among academia, industry, and policymakers to establish standardized databases, implement green manufacturing strategies, and integrate artificial intelligence for process optimization.

Looking forward, biomass-derived carbon materials could play a key role in future energy storage systems if these structural, technological, and regulatory barriers are systematically addressed. Although recent studies demonstrate promising laboratory-scale performance, their commercial deployment remains limited due to feedstock variability, challenges in scalable production, and the absence of standardized testing protocols.

These limitations could be mitigated through the adoption of green activation methods, AI-based optimization strategies, and integration within a circular-economy framework. Nevertheless, translating this potential into practical applications requires coordinated efforts in materials engineering, process automation, and policy support to ensure alignment between environmental sustainability and economic feasibility.

The future development of biomass-derived supercapacitor electrodes as next-generation sustainable energy storage materials will depend on the convergence of sustainability-driven innovation and industrial standardization.

## 5. Conclusions

Interest in sustainable energy storage has increasingly focused on biomass-derived carbons, utilizing food waste and other lignocellulosic residues to produce high-performance, environmentally friendly supercapacitor electrodes. The use of waste biomass not only addresses environmental challenges but also offers a cost-effective route toward scalable and sustainable energy storage devices. This approach exemplifies the circular economy concept, in which food waste is converted into value-added materials, thereby supporting the advancement of renewable energy technologies.

This review highlights the potential of biomass-based carbon materials using food waste-derived precursors as a key pathway to transform the existing energy storage technologies, especially supercapacitors, to be sustainable. The results indicate that food waste and other lignocellulosic residues and agro-food by-products are also promising low-cost, renewable, and plentiful feedstocks, which can be easily transformed into high-performance electrode materials. These biomass carbons exhibit competitive specific capacitance values (up to 520 F g^−1^), large surface areas of more than 1500 m^2^ g^−1^, and great cycling stability (approaching 95% retention with 10,000 cycles), competing with or exceeding conventional activated carbons, graphene and carbon nanotubes. Significantly, the heteroatom doping (N, O, S, P) of most food waste contributors increases pseudocapacitive behaviour, which can play a role in increasing energy density in addition to the wettability factors crucial in practice. Despite their promising electrochemical properties, the reproducibility of biomass-derived precursors remains a critical challenge for large-scale applications. Variations in precursor composition, seasonal availability, and processing conditions can significantly affect the structural properties of the resulting carbon materials.

The valorization of food waste aligns with the principles of the circular economy, where environmental sustainability is achieved through the reduction in landfill burden, mitigation of greenhouse gas emissions, and decreased reliance on fossil-derived materials. Biomass-derived carbons are expected to enhance the commercial viability of supercapacitors by providing not only environmentally sustainable but also economically competitive solutions for large-scale manufacturing.

Furthermore, the integration of machine learning and AI-driven optimization can accelerate materials development and performance optimization. Future studies should focus on addressing key challenges related to process scalability, standardization, and life-cycle assessment. Sustainable activation strategies, process automation, and policy frameworks should be developed to bridge the gap between laboratory-scale achievements and industrial implementation. With coordinated efforts across academia, industry, and policymaking, biomass-derived supercapacitors are expected to play a pivotal role in the transition toward sustainable energy systems, leveraging waste valorization to meet the demands of a low-carbon future.

## Figures and Tables

**Table 1:** Comparative summary of charge storage mechanisms in supercapacitors.

No	Feature	Electric Double-Layer Capacitance (EDLC)	Pseudo-Capacitance	Critical Hybrid Implications	Ref.
1	Nature of Process	Non-faradaic (electrostatic charge separation at electrode–electrolyte interface)	Faradaic (fast and reversible redox reactions at or near electrode surface)	Hybrid supercapacitors often combine both to enhance energy and power performance	[37]
2	Charge Storage Mechanism	Ion adsorption at the electrode surface forming a Helmholtz double layer (inner and diffuse layers)	Redox reaction, ion intercalation, or surface electrosorption processes involving charge transfer	The combination enables dual storage pathways, improving both specific capacitance and stability in	[45]
3	Electrode Materials	Carbon-based materials (activated carbon, graphene, carbon nanotubes, carbon aerogels)	Transition metal oxides (MnO_2_, Co_3_O_4_, Nb_2_O_5_, NiO) and conducting polymers (polyaniline, polypyrrole)	MXene and graphene composites enable hybrid EDLC–pseudocapacitive behavior	[43,44]
4	Charge Transfer	No charge transfer; purely electrostatic	Involves electron transfer through redox reactions	Hybrid electrodes balance electrostatic and faradaic contributions	[46]
5	Capacitance Range (F g^−1^)	100–300	500–1500	Hybrid systems can reach 400–1000 F g^−1^ depending on design	[47]
6	Energy Density (Wh kg^−1^)	5–15	60–100	Hybrids achieve intermediate energy densities (20–60 Wh kg^−1^)	[42]
7	Power Density (kW kg^−1^)	5–15	1–5	Hybrids maintain high power (~10 kW kg^−1^) with improved energy	[38]
8	Cycle Life	>10^6^ cycles (excellent reversibility)	10^3^–10^5^ cycles (degradation due to volume changes)	Hybrid systems enhance cycle life through stable interfaces	[40]
9	Kinetic Behavior	Fast ion adsorption/desorption (diffusion-limited)	Surface-controlled redox kinetics (reaction-limited)	Optimized pore structures and nanostructured composites minimize kinetic limitations	[39]
10	Typical Applications	Regenerative braking, UPS systems, portable electronics	Grid storage, flexible electronics, and hybrid vehicles	Hybrid designs are promising for high-performance sustainable energy storage	[41]

**Table 3:** Chemical composition of food waste biomass and its influence on carbon yield, porosity, conductivity, and heteroatom doping.

No	Component	Content in Food Waste (wt%)	Structural/Functional Features	Influence on Carbon Properties	Remarks	Ref.
1	Cellulose	30–50	Linear polysaccharide rich in hydroxyl groups	Forms ordered carbon microstructures with high microporosity and specific surface area (>1000 m^2^ g^−1^); improves ion diffusion	Requires controlled pyrolysis to avoid pore collapse	[66,67,73]
2	Hemicellulose	20–30	Branched polysaccharide with amorphous structure	Generates volatile gases leading to mesoporous formation; enhances surface reactivity	Causes lower carbon yield due to high volatility	[65,67,74]
3	Lignin	10–30	Highly aromatic and thermally stable polymer	Increases carbon yield and graphitization degree; improves electrical conductivity	Difficult to decompose; may produce non-uniform carbon	[68,69]
4	Proteins	5–15	Peptide chains with N, S, and O functional groups	Introduces N/S dopants improving pseudocapacitance, wettability, and electron transport	Excessive content may cause pore blockage or unstable nitrogen forms	[70,75]
5	Lipids	2–10	Long-chain hydrocarbons with ester groups	Enhance carbon hydrophobicity and provide self-activation during pyrolysis	May reduce surface polarity if not well oxidized	[72,73]
6	Minerals (K, Ca, Mg, Na)	3–8	Inorganic salts acting as natural catalysts	Promote in situ activation, pore development, and increased surface roughness	High mineral content may cause ash formation, reducing purity	[69,71,76]

**Table 4:** Assessments of biomass-derived carbon materials (BDCMs) for supercapacitor applications.

No	Source	Technique	Advantage	Economic Advantage	Electrochemical Performance	Ref.
1	Food waste (mixed)	Hydrothermal carbonization	↓ ~65% GHG emissions; eliminates landfill leachate	Near-zero feedstock cost; decentralized processing	210–245 F g^−1^ at 1 Ag^−1^	[98]
2	Lignocellulosic residues (wood, husk)	Physical/chemical activation	Low life-cycle CO_2_ footprint; valorization of agri-waste	40–50% lower cost than fossil-based carbon	250–280 F g^−1^; high-rate stability	[99]
3	Agri-food waste (tea, fruit peels)	Pyrolysis + KOH activation	Carbon sequestration; reduced methane release	Minimal pretreatment; scalable fabrication	220–260 F g^−1^; 38 Whkg^−1^ energy density	[100]
4	Lignocellulosic agri-waste	Engineered biochar/hydrochar production	CO_2_ offset > 1.5 t per t biomass; circular economy loop	High yield; reusable activating agents	200–240 F g^−1^; >95% retention after 5000 cycles	[101,102]
5	Food waste biochar composites	Controlled carbonization + doping	Enhanced waste valorization; lower emission intensity	60% lower synthesis energy demand	260–310 F g^−1^; improved energy density ~42 Wh kg^−1^	[103,104]

↓ = Reduction.

**Table 5:** Comparative summary of biomass-derived carbon and conventional electrode materials for supercapacitor applications.

No	Type of Material	Performance (Specific Capacitance/Surface Area)	Applications	Importance	Disadvantages	Ref.
1	Biomass-derived carbon materials	200–350 F g^−1^; surface area up to 1400 m^2^ g^−1^; energy density 10–25 Wh kg^−1^	Supercapacitor electrodes, CO_2_ capture, and multifunctional energy storage	Renewable, low-cost, tunable porosity, heteroatom-doped, and environmentally sustainable	Limited conductivity compared to metal oxides; dependent on precursor uniformity	[110]
2	Metal-based materials (Ni, Co, Fe, Mn)	500–900 F g^−1^; high redox activity	Pseudocapacitors, hybrid supercapacitors	High electrical conductivity and specific capacitance	Expensive, prone to aggregation and oxidation; low cyclic stability	[106,109]
3	Metal oxide-based materials (MnO_2_, Co_3_O_4_, NiO)	600–1200 F g^−1^; good pseudocapacitance	High-performance energy storage and electrochemical catalysis	High theoretical capacitance and electrochemical activity	Poor rate capability, structural instability upon cycling	[107,111]
4	Clay-based materials (kaolinite, montmorillonite, bentonite)	100–300 F g^−1^; moderate ion transport	Flexible and solid-state supercapacitors	Naturally abundant, low-cost, and environmentally benign	Low conductivity and surface area; needs modification or composites	[108]
5	Composite materials (carbon/metal oxide, polymer/oxide hybrids)	400–1000 F g^−1^; enhanced cycling stability > 90% after 5000 cycles	Multifunctional, structural, and flexible supercapacitors	Synergistic effects combine conductivity and pseudocapacitance; high performance	Complex fabrication, costly precursors, recyclability issues	[112]

**Table 6:** Surface modification strategies for enhanced electrochemical performance of biomass-derived carbon materials.

No	Strategy/Material Type	Structural Features	Activation Method	Doping Materials	Mechanistic Enhancement	Specific Capacitance (F g^−1^)	Energy Density (Wh kg^−1^)	Ref.
1	Hierarchical porous carbon (HPC) from biomass	Interconnected micro–meso–macro pores; high ion-accessibility	Chemical activation (KOH)		Fast ion diffusion; double-layer formation	310	42	[117]
2	Food-waste-derived activated carbon	Ultrahigh surface area (~2100 m^2^ g^−1^); tunable porosity	CO_2_ + KOH dual activation		Enhanced electrolyte penetration; high surface charge accumulation	285	38	[118,119]
3	N-doped porous carbon	Graphitic-N, pyridinic-N sites; improved electron mobility	Ammonia annealing/urea doping	N	Increased conductivity and redox contribution	350	45	[127]
4	N,S co-doped carbon (PEDOT-assisted)	Defect-rich surface; conductive polymer network	In situ polymerization with PEDOT	N, S	Synergistic faradaic sites; enhanced wettability and durability	420	48	[124,128]
5	Multi-heteroatom-doped carbon (B/N/P/S)	High defect density; strong surface polarity	Biomass precursor + multi-step carbonization	B, N, P, S	Multiple redox-active sites; boosted ion adsorption energy	460	50	[124,129]
6	NiO/Co_3_O_4_-modified porous carbon	Nanostructured oxide coating on carbon matrix	Impregnation–calcination	NiO, Co_3_O_4_	Redox pseudocapacitance + enhanced electron transfer	1020	58	[130,131]
7	Ni-MOF@rGO composite	MOF nanoparticles anchored on rGO; high conductivity	MOF growth + reduction process	Ni, O	Dual EDLC–pseudocapacitive behavior; fast ion transport	2150	62	[124]
8	Co-MOF-sulfide hybrid	Hierarchical porous structure; high surface redox activity	Sulfidation of Co-MOF precursor	Co, S	Strong redox kinetics; long-term cycling stability	>2600	65	[130]
9	MXene/polymer–carbon hybrid	Conductive framework; high interfacial contact area	Blending + in situ polymerization	Ti, C, N	Enhanced charge mobility and structural resilience	870	55	[125]

**Table 7:** Food waste-derived carbons and conventional carbon materials for supercapacitor applications.

No	Carbon Material Type	Surface Area (m^2^ g^−1^)	Conductivity (S m^−1^)	Specific Capacitance (F g^−1^)	Energy Density (Wh kg^−1^)	Cycle Stability (%)	Cost/Scalability	Reference
1	Food waste-derived carbon	1200–2800	10^2^–10^3^	350–520	35–50	93–98 (10,000 cycles)	Very low/Highly scalable	[132,134,135]
2	Activated carbon	800–2500	10^1^–10^2^	200–350	20–35	90–95 (5000–8000 cycles)	Low/Commercially scalable	[135]
3	Graphene	1500–2600	~10^4^	300–600	40–60	90–97 (10,000 cycles)	High/Limited scalability	[136,137]
4	Carbon nanotubes (CNTs)	500–1500	10^3^–10^4^	200–450	30–45	92–96 (10,000 cycles)	Very high/Difficult to scale	[133,138]

## Data Availability

No new data were created or analyzed in this study. Data sharing is not applicable to this article.
